# Role of miR-100-5p and CDC25A in breast carcinoma cells

**DOI:** 10.7717/peerj.12263

**Published:** 2022-01-03

**Authors:** Xiaoping Li, Yanli Ren, Donghong Liu, Xi Yu, Keda Chen

**Affiliations:** 1 Faculty of Medicine, Macau University of Science and Technology, Macau, China; 2 Shulan International Medical College, Zhejiang Shuren University, Hangzhou, Zhejiang province, China; 3 State Key Laboratory for Diagnosis and Treatment of Infectious Diseases, National Clinical Research Center for Infectious Diseases, The First Affiliated Hospital, Zhejiang University School of Medicine, Hangzhou, Zhejiang province, China; 4 Department of Laboratory Medicine, Hangyan hospital of Wenzhou Medical University, Taizhou First People’s Hospital, Taizhou, Zhejiang, China

**Keywords:** MiR-100-5p, CDC25A, Breast carcinoma, Proliferation, Migration and invasion

## Abstract

**Objective:**

To inquiry about mechanism of miR-100-5p/CDC25A axis in breast carcinoma (BC), thus offering a new direction for BC targeted treatment.

**Methods:**

qRT-PCR was employed to explore miR-100-5p and CDC25A mRNA levels. Western blot was employed for detecting protein expression of CDC25A. Targeting relationship of miR-100-5p and CDC25A was verified by dual-luciferase assay. *In vitro* experiments were used for assessment of cell functions.

**Results:**

In BC tissue and cells, miR-100-5p was significantly lowly expressed (*P* < 0.05) while CDC25A was highly expressed. Besides, miR-100-5p downregulated CDC25A level. miR-100-5p had a marked influence on the prognosis of patients. The forced miR-100-5p expression hindered BC cell proliferation, migration and invasion, and facilitated cell apoptosis. Upregulated miR-100-5p weakened promotion of CDC25A on BC cell growth.

**Conclusion:**

Together, these findings unveiled that CDC25A may be a key target of miR-100-5p that mediated progression of BC cells. Hence, miR-100-5p overexpression or CDC25A suppression may contribute to BC diagnosis.

## Introduction

Breast carcinoma (BC) accounts for 7–10% of all solid malignancies (Feng et al., 2015). At present, the incidence and mortality rate of BC are increasing annually owing to the difficulty in the early diagnosis of BC caused by the complexity of endocrine system (Al-Hajj et al., 2003). Great improvements have been made in prognosis of BC patients in recent years. However, five-year survival rate of patients is still low, and postoperative malignant behaviors of BC cells are main causes of the disease (Anastasiadi et al., 2017). Therefore, it is of importance to make in-depth inquiry about mechanism of BC metastasis and actively search the target gene for the targeted therapy of BC.

MicroRNAs (miRNAs) are evolutionarily conserved (Gangaraju & Lin, 2009). miRNA expression is considered to be a part of multiple normal biological processes (Ambros, 2003). Studies indicated that abnormity of miRNAs exists in several malignancies including BC, and affects the malignant behaviors of cancer cells (Yerukala Sathipati & Ho, 2020; Volovat et al., 2020). For example, miR-520c-3p was reported to negatively modulate epithelial-mesenchymal transition (EMT) *via* targeting Interleukin-8 (IL-8) to hamper BC cell malignant progression (Tang et al., 2017). miR-485-5p can downregulate transmembrane glycoprotein Mucin 1 (MUC1) to constrain BC cell proliferation, invasion, migration, and to hasten cell apoptosis (Wang et al., 2020). miR-539 upregulation represses BC progression *via* targeting specificity protein 1 (SP1), indicating that miR-539 is a possible target for diagnosis of BC (Cai et al., 2020). Taken together, these findings evince that miRNAs are important in BC. miR-100-5p as an essential member of miR-100 family is highly conserved (Nabavi et al., 2017). miR-100 is aberrantly expressed in diverse tumors, including esophageal squamous carcinoma (Zhou et al., 2016), glioblastoma (Luan et al., 2015), gastric cancer (Shi et al., 2015) and renal cell carcinoma (Chen et al., 2017). Nonetheless, exploration of miR-100-5p function in BC cell metastasis necessitates extensive research.

CDC25 family members can regulate cell cycle (Brenner et al., 2014). CDC25 modulates cell cycle progression by hindering cyclin-dependent kinases (CDKs) phosphorylation, so as to activate the CDK complexes (Blomberg & Hoffmann, 1999). The high CDC25A expression has been found in different cancer types, and overexpression of CDC25A presents in approximately 50% of BC cases and implicates poor prognosis (Cangi et al., 2000). A previous study denoted that CDC25A regulates stem cell proliferation *via* targeting Sirtuin6 (SIRT6) in colorectal cancer (Liu et al., 2018). miR-365 strengthens radiosensitivity of non-small cell lung cancer (NSCLC) cells *via* targeting CDC25A (Li et al., 2019). miR-98-5p hinders osteosarcoma progression *via* targeting CDC25A (Liu & Cui, 2019). However, the regulatory mechanism of CDC25A in BC has not been explored.

By bioinformatics methods, we identified low miR-100-5p expression in BC. Modulatory role of miR-100-5p was investigated in BC *via* experiments, disclosing that miR-100-5p targeted CDC25A to restrain BC cell progression. Our investigation contributes to mechanistic understanding of BC progression, thereby providing targeted therapy for BC with novel theoretical basis.

## Materials and Methods

### Bioinformatics methods

Mature miRNA (104 normal samples, 1,103 tumor samples) and mRNA (113 samples, 1,109 samples) expression data of TCGA-BRCA dataset were obtained from TCGA (https://portal.gdc.cancer.gov/) database. Expression analysis and survival analysis were carried out on the target miRNA according to the obtained data. Differential mRNAs (DEmRNAs) were got through differential analysis by using “edgeR” package (—logFC—>2.0 and *padj*<0.01). Three databases miRDB, starBase, and mirDIP were used to predict target mRNAs of miR-100-5p. Candidate mRNAs were got by overlapping up-regulated DEmRNAs and predicted mRNAs of miR-100-5p, among which a mRNA having the highest correlation coefficient was picked as the objective of study.

### Cell culture

Human breast epithelial cell line MCF-10A (BNCC337734), human BC cell lines T47D (BNCC339607), MDA-MB-231 (BNCC339911), MCF-7 (BNCC100137) and BT-474 (BNCC101989) were bought from BeNa Culture Collection (Beijing, China) and cultured at 37 °C with 5% CO_2_. Information of culture mediums:

MDA-MB-231, MCF-7, T47D cell lines: DMEM with 10% fetal bovine serum (FBS) and 100 U/mL penicillin/streptomycin;

MCF-10A, BT-474 cell lines: RPMI-1640 medium with 10% FBS.

### Cell transfection

miR-100-5p mimic (miR-mimic), mimic NC (miR-NC), pcDNA3.1 (oe-NC), and pcDNA3.1-CDC25A plasmid (oe-CDC25A) were acquired from GenePharma Company (Shanghai, China). Cell transfection recommended lipofectamine 2000 (Invitrogen, Carlsbad, CA, USA).

### qRT-PCR

Total RNA was extracted from cells by TRIzol kit (Invitrogen, Carlsbad, CA, USA) according to instructions. Concentration was assayed using NanoDrop 2000 system (Thermo Fisher Scientific, Inc., Waltham, MA, USA). cDNA was generated by reversely transcribing miRNA with miScript IIRT kit (Qiagen, USA) and mRNA with PrimeScript RT Master Mix (Takara, Dalian, P.R. China) according to protocols. qRT-PCR was completed on Applied Biosystems^®^ 7500 Real-Time PCR Systems (Thermo Fisher Scientific, Waltham, MA) with miScript SYBR Green PCR Kit (Qiagen, Germany) and SYBR^®^ Premix Ex Taq TM II (Takara Bio Inc., Shiga, Japan). PCR conditions were as follows: 95 °C 10 s, 60 °C 20 s, 72 °C 20 s, 45 cycles. 2^−ΔΔCt^ method was utilized for data analyzing. GAPDH and U6 were applied as endogenous controls. Primer sequences were exhibited in Table 1.

**Table 1:** Primer sequences used in qRT-PCR.

Gene	Primer sequence (5′→ 3′)	
miR-100-5p	F: AACCCGTAGATCCGAACTTGTG	
U6	F: CTCGCTTCGGCAGCACA	R: ACGCTTCACGAATTTGCGT
CDC25A	F: GAGGAGTCTCCACCTGGAAGTACA	R: GCCATTCAAAACAGATGCCATAA
GAPDH	F: GACCTGACCTGCCGTCTA	R: AGGAGTGGGTGTCGCTGT

### CCK-8

Forty-eight h after transfection, BC cell line MCF-7 suspended in DMEM plus 10% FBS was added into 96-well plates (2  × 10^3^ cells/well). Ten µL CCK-8 solution (CK04; Dojindo Laboratories, Kumamoto, Japan) was added at 24, 48, 72 and 96 h for 2 h of incubation under standard conditions. Absorbance value at 450 nm was read at designated time points.

### Clonogenic assay

Cells in varying transfection groups were treated with 0.25% trypsin, inoculated into 6-well plates (4  × 10^2^ cells/well) and incubated in DMEM with 10% FBS for 2 weeks. Colonies were subjected to fixation with 95% methanol, 10 min of staining with 0.1% crystal violet, and rinsing with PBS, followed by cell colony counting.

### Wound healing assay

After transfection, 1  × 10^5^ BC cells (MCF-7) were placed into 6-well plates, and a pipette tip (200 µL) was employed to make a scratch through center of each well when cells grew to 80% confluence. Dispersed cells were discarded. Fresh mediums were employed to continuously culture the remaining cells. The migrated cells were observed and pictured at 0 and 24 h. Image J software was used for analysis. Migration width = wound healing width at 24 h –wound healing width at 0 h.

### Transwell invasion assay

First, 9.6 mg/ml Matrigel matrix (356234, BD Company, USA) diluted with serum-free medium at 1:8. Fifty µL of dilution was added to the Transwell upper chambers for 30 min. After transfected for 48 h, BC cells were suspended in 200 µl serum-free DMEM (1  × 10^5^ cells/mL) and were filled into the upper chamber, while medium with 15% FBS was filled into lower chamber. After being cultured for 48 h at 37 °C, cells that did not pass membranes were removed using a cotton swab, while cells in lower chamber were subjected to 0.1% crystal violet for staining. A microscope was implicated to random 4 fields, and invaded cells were counted and photographed.

### Western blot

Cells were lysed by radioimmunoprecipitation assay buffer (RIPA; Sigma-Aldrich), and protein concentration was assessed by Pierce BCA (Thermo, USA) protein assay kit. Total proteins were isolated by 10% SDS-PAGE and transferred onto polyvinylidene fluoride (PVDF) membranes (Sigma-Aldrich). After blocking, membranes were cultivated overnight with primary antibodies, including rabbit anti-CDC25A and rabbit anti-GAPDH. Subsequently, PBS + 0.1% Tween-20 (PBST) was taken to rinse membranes 3 ×10 min. Afterward, secondary antibody goat anti-rabbit IgG H&L (HRP) (ab205718) was incubated with membranes at room temperature for 1 h, and then membranes were rinsed with PBST 3 ×10 min. All protein bands were visualized by chemiluminescence reaction (Bio-Rad, Hercules, CA, USA), followed by analysis by Image Lab (Bio-Rad). All antibodies were from Abcam (China).

### Dual-luciferase assay

Vectors psiCHECK (Sangon Co., LTD, Shanghai, China) fused with mutant-type (MUT) and wild-type (WT) CDC25A 3′ UTR were generated. MCF-7 cells were planted onto 48-well plates for 24 h of culture at 37 °C. miR-100-5p mimic/mimic NC and CDC25A-psiCHECK WT/MUT were co-transfected into cells. Forty-eight h later, the luciferase activity was assayed by Dual-Luciferase Reporter Assay System (Promega, Fitchburg, WI, USA).

### Flow cytometry

BC cells in logarithmic growth phase were transfected. Forty-eight h later, cells were collected. Cell suspension (100 µL) was incubated 15 min in dark with Annexin-V-FITC (5 µL, KeyGen Biotech, Nanjing, China), and then propidium iodide (2.5 µL, PI) was utilized for cell staining. FACS Calibur (BD Biosciences, San Jose, CA, USA) was adopted to measure cell apoptosis. Flowjo software (Tree Star Corp, San Carlos, CA, USA) was recommended for data analysis.

### Statistical analysis

All data were processed by GraphPad Prism 6.0 (La Jolla, CA). Each experiment was repeated in triplicate, including 3 technical replicates and 3 biological replicates. The results were presented by mean ± standard deviation (SD). To compare differences among three or more groups, one-way analysis of variance, followed by Bonferroni test was used. Student’s *t*-test was used for significance test between the two groups. *P* < 0.05 was accepted as significant.

### Result

### miR-100-5p is lowly expressed in BC tissue and cells

**Figure 1:**
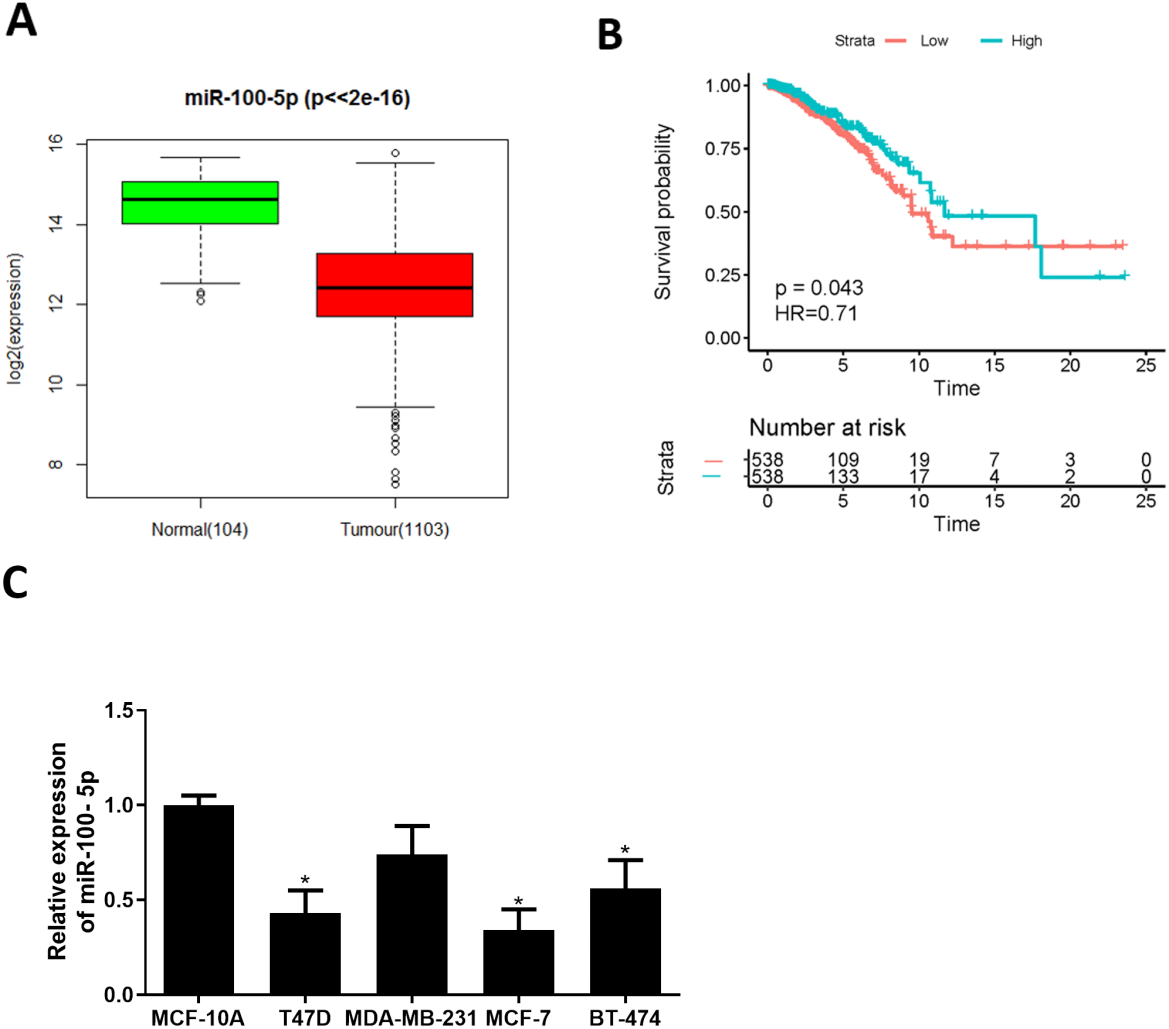
miR-100-5p is lowly expressed in BC cells. (A) Box plots of miR-100-5p (normal sample: green, 104; tumor sample: red, 1103); (B) Survival curves of miR-100-5p. The x-axis refers to time (years), y-axis refers to survival rate; (C) miR-100-5p level in MCF-10A (human breast epithelial cell line) and T47D, MDA-MB-231, MCF-7 and BT-474 (human BC cell lines) assayed via qRT-PCR; * *P* < 0.05; Experiment in Fig. C was repeated 3 times, including 3 technical replicates and 3 biological replicates, and representative figures were selected.

Bioinformatics analysis was employed to analyze miRNA expression data in TCGA-BRCA dataset. miR-100-5p was conspicuously down-regulated in BC tissue (Fig. 1A), while survival analysis suggested that low miR-100-5p level was related to poor prognosis (Fig. 1B). A lot of studies also manifest that miR-100 is essential in regulating pathogenesis of BC (Gebeshuber & Martinez, 2013; Chen et al., 2014; Gong et al., 2015; Jiang et al., 2016). Therefore, miR-100-5p was chosen as the miRNA of interest. Thereafter, qRT-PCR result disclosed that miR-100-5p was considerably reduced in T47D, MCF-7, and BT-474 cells (*P* < 0.05) (Fig. 1C). Thus, we concluded that miR-100-5p was decreased in BC. MCF-7 cell line with the lowest miR-100-5p level was selected for future detections.

### The forced miR-100-5p expression hinders BC cell malignant progression

**Figure 2:**
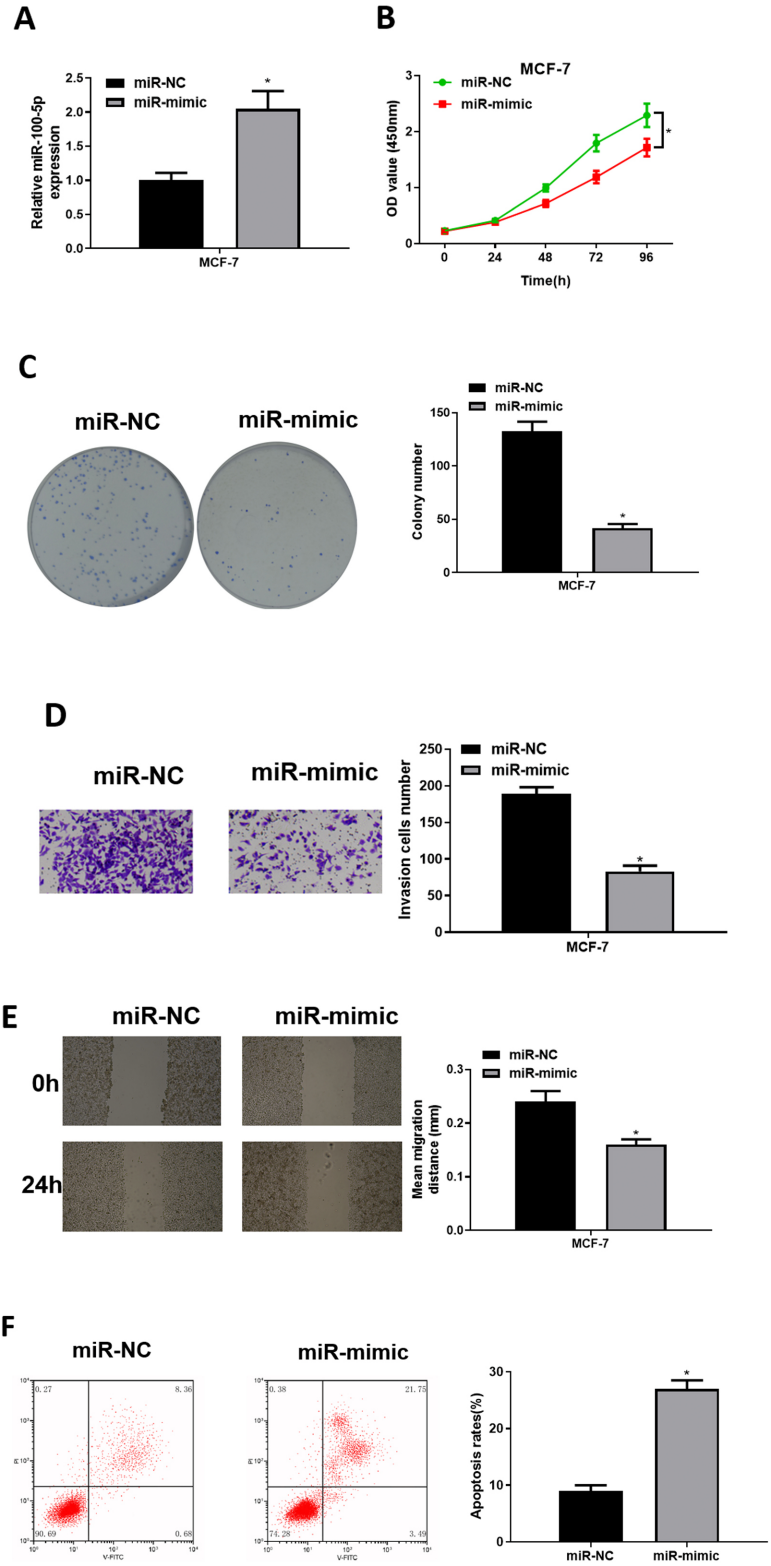
The forced miR-100-5p expression hinders BC cell proliferation, migration and invasion, and fosters cell apoptosis. (A) Transfection efficiency of miR-100-5p mimic in MCF-7 cells measured via qRT-PCR; (B) cell viability, (C) clonogenic, (D) cell invasive property, (E) cell migratory property; (F) cell apoptosis in different groups; * *P* < 0.05; Experiment in Figure 2 was repeated three times, including three technical replicates and three biological replicates, and representative figures were selected.

To explore role of miR-100-5p in BC, mimic NC group and miR-100-5p mimic groups were designed for verification. qRT-PCR was employed to assay transfection efficiency into MCF-7, presenting that miR-100-5p was notably increased in miR-100-5p mimic group (*P* < 0.05) (Fig. 2A). CCK-8 assay result showed that effect of enforced miR-100-5p expression potently suppressed MCF-7 cell proliferation (*P* < 0.05) (Fig. 2B). As unveiled *via* clonogenic assay, overexpressing miR-100-5p highly inhibited cell clonogenic capacity (*P* < 0.05) (Fig. 2C). Results of Transwell and wound healing assays discovered that these properties were remarkably inhibited after miR-100-5p expression was enforced (*P* < 0.05) (Figs. 2D and 2E). Flow cytometry indicated that overexpressing miR-100-5p fostered MCF-7 cell apoptosis (*P* < 0.05) (Fig. 2F). It was displayed that miR-100-5p overexpression inhibited BC cell proliferative, migratory, invasive properties, and hastened cell apoptosis.

### miR-100-5p inhibits CDC25A in BC cells

**Figure 3:**
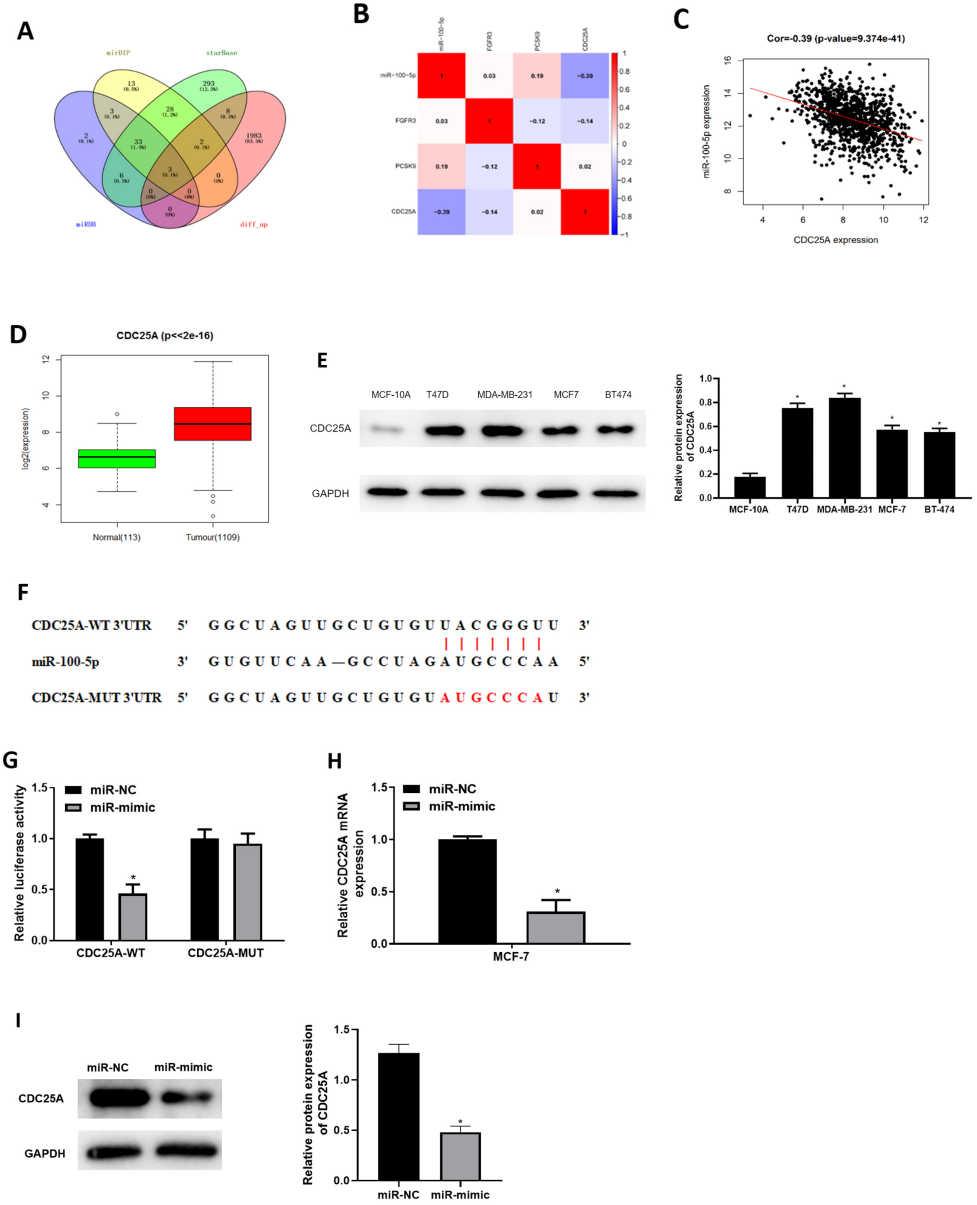
miR-100-5p targets and reduces CDC25A level in BC cells. (A) Venn diagram of up-regulated DEmRNAs and predicted downstream genes of miR-100-5p; (B) Pearson correlation analysis of miR-100-5p and predicted genes CDC25A, FGFR3 and PCSK9; (C) Pearson correlation analysis of miR-100-5p and CDC25A; (D) box plots of CDC25A level (green refers to normal and red refers to tumor; normal sample: 113; tumor sample: 1109); (E) Western blot assessed CDC25A expression in MCF-10A, T47D, MDA-MB-231, MCF-7, and BT-474; (F) the binding sequences of miR-100-5p and CDC25A; (G) binding of miR-100-5p and CDC25A validated through dual-luciferase assay; (H–I) CDC25A mRNA and protein levels in MCF-7 cells; * *P* < 0.05; Experiments in Figure E, G–I was repeated three times, including three technical replicates and three biological replicates, and representative figures were selected.

Three databases miRDB, mirDIP and starBase were applied for predicting target mRNAs of miR-100-5p. Three candidates obtained from overlap of up-regulated DEmRNAs and predicted mRNAs of miR-100-5p (Fig. 3A) were analyzed by correlation analysis with miR-100-5p, and CDC25A was applied as the target mRNA due to its highest correlation coefficient (Figs. 3B and 3C). CDC25A was found to be greatly up-regulated in BC tissue (*P* < 0.05) (Fig. 3D). In BC cells, CDC25A mRNA and protein expression levels were relatively high (Fig. 3E). We also disclosed complementary binding sites of miR-100-5p and FCDC25A. Bioinformatics analysis revealed binding of miR-100-5p and CDC25A (Fig. 3F). Dual-luciferase assay manifested that overexpressed miR-100-5p suppressed luciferase activity of CDC25A-WT (*P* < 0.05) but had no influence on that of CDC25A-MUT (Fig. 3G). qRT-PCR was then implemented to detect CDC25A mRNA level in MCF-7 cell line, showing a decreased trend of CDC25A mRNA level in miR-100-5p-mimic group (*P* < 0.05) (Fig. 3H). Western blot uncovered that CDC25A protein level was noticeably reduced after miR-100-5p was enforced (*P* < 0.05) (Fig. 3I). It was revealed that miR-100-5p suppressed CDC25A in BC cells.

### miR-100-5p targets CDC25A to repress cell proliferation, migration, invasion, and induce cell apoptosis in BC

**Figure 4:**
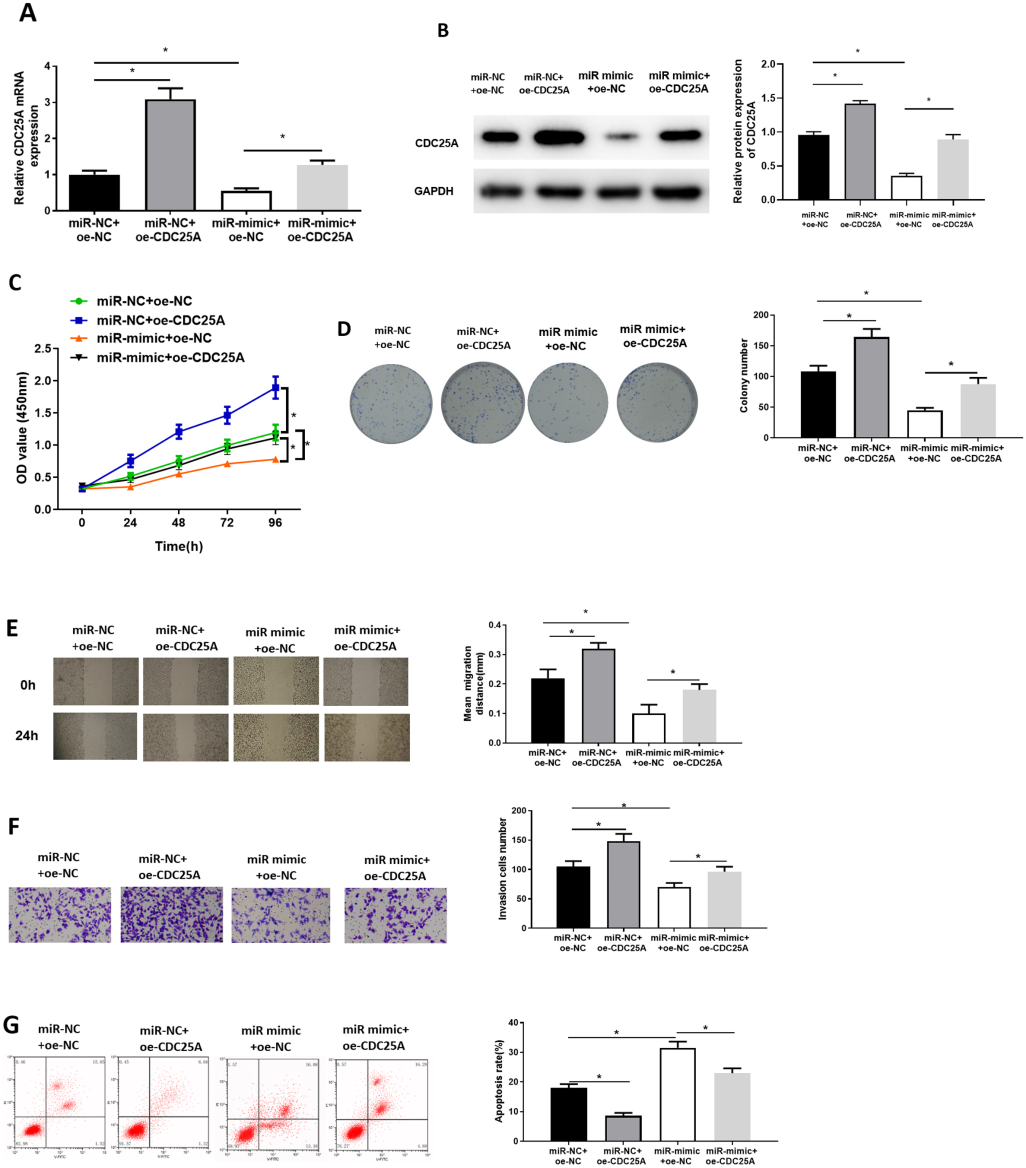
miR-100-5p hampers cell malignant behaviors by targeting CDC25A in BC. The (A) mRNA and (B) protein levels of CDC25A in transfected cells assayed through qRT-PCR and western blot; (C) Proliferation, (D) clonogenic, (E) migration and (F) invasion properties of MCF-7 cells in different treatment groups assayed through CCK8, clonogenic , wound healing (40×) and Transwell assays (100×), and (G) flow cytometry was carried out for determination of cell apoptotic rate; * *P* < 0.05; Each experiment was repeated three times, including three technical replicates and three biological replicates, and representative figures were selected.

To investigate impact of miR-100-5p targeting CDC25A on malignant behaviors of BC, we established four groups: miR-NC+oe-NC (negative control group), miR-NC+oe-CDC25A (CDC25A overexpression group), miR-mimic+oe-NC (miR-100-5p overexpression group), and miR-mimic+oe-CDC25A (simultaneous enforced expression of miR-100-5p and CDC25A group). qRT-PCR and western blot result found that CDC25A mRNA and protein levels were markedly up-regulated in oe-CDC25A group (*P* < 0.05), and notably downregulated when miR-100-5p was overexpressed (*P* < 0.05). In comparison to miR-mimic+oe-NC group, CDC25A was upregulated in miR-100-5p mimic+oe-CDC25A group (*P* < 0.05) (Fig. 4A and 4B), which clarified that CDC25A was down-regulated by miR-100-5p. The results of CCK-8 and clonogenic assays illustrated that overexpressing CDC25A potently increased BC cell proliferation ability (*P* < 0.05), while upregulating miR-100-5p could notably suppress this ability(*P* < 0.05). In comparison to miR-mimic+oe-NC group, cell proliferative ability increased when CDC25A and miR-100-5p were simultaneously overexpressed (*P* < 0.05) (Figs. 4C and 4D). Subsequently, wound healing and Transwell revealed that overexpressing CDC25A potently increased BC cell migratory and invasive properties (*P* < 0.05), while upregulating miR-100-5p could remarkably repress these abilities (*P* < 0.05). In comparison to miR-mimic+oe-NC group, these abilities were clearly enhanced after CDC25A and miR-100-5p were simultaneously overexpressed (*P* < 0.05) (Figs. 4E and 4F). Furthermore, flow cytometry also substantiated that apoptotic rate of MCF-7 cells was considerably reduced in oe-CDC25A group (*P* < 0.05), while miR-100-5p upregulation could dramatically facilitate cell apoptosis (*P* < 0.05). In comparison to miR-mimic+oe-NC, cancer cell apoptotic rate was restrained after CDC25A and miR-100-5p were simultaneously overexpressed (*P* < 0.05) (Fig. 4G). In conclusion, we manifested that miR-100-5p hindered BC cell proliferation, migration, invasion, and hastened cell apoptosis by reducing CDC25A expression.

### Discussion

With 1.3 million cases increasing annually, BC is a symbolic cancer in female’s daily life (Qin & Liu, 2019). A recent study has illustrated that miRNAs might be potential therapeutic targets for BC (Luan et al., 2017). miRNAs are regarded as effective modulators of cell proliferation, metastasis, translation and tumorigenesis (Zhou et al., 2017). There is a correlation between miRNAs and mRNAs that makes specific cure of certain cancers possible at molecular level (Zhou et al., 2013).

Present studies disclosed that miR-100-5p is aberrantly expressed in several cancers, modulating cancer cell malignant behaviors. For instance, miR-100-5p is lowly expressed in prostate cancer cells, and it down-regulates mTOR to hamper progression of prostate cancer cells (Ye, Li & Wang, 2020). In chordoma tissue, miR-100-5p is down-regulated, while enforced miR-100-5p expression can hinder chordoma growth, and it can partially hinder chordoma cell malignant behaviors *via* EMT suppression (Zhang et al., 2020). Wang, Tao & Bian (2021) unveiled that miR-100-5p may be a new biomarker for patients with skin melanoma, and it may be related to survival time of patients. Moreover, Annalisa et al. (Petrelli, Bellomo et al. 2020) elucidated that miR-100 can be used as a novel biomarker for patients suffering luminal BC. Nonetheless, research on role and modulatory mechanism of miR-100-5p in BC is in high unmet need. In this study, we first carried out bioinformatics analysis, revealing low miR-100-5p expression in BC tissue. qRT-PCR detection also found that miR-100-5p was decreased in BC cells. Additionally, we noted that overexpression of miR-100-5p inhibited progression of MCF-7 cells as well, indicating that miR-100-5p had a cancer-suppressive effect on BC cells, congruous with earlier investigations.

To further elucidate modulatory mechanism of miR-100-5p in progression of BC, CDC25A was predicted to be a target of miR-100-5p, which was unveiled to be highly expressed in BC through bioinformatics analysis. CDC25A is a key regulatory factor in progression of cell cycle and checkpoint response (Jin, 2011). miR-365 facilitates radio-sensitivity of NSCLC cells *in vitro* and *in vivo* by targeting CDC25A (Ding et al., 2019). miR-122-5p has an inverse modulatory impact on CDC25A expression, which is conducive to the improvement of the prognosis of cervical cancer patients (Ding et al., 2019). miR-449a targets CDC25A to noticeably reduce cell proliferation and invasion, while inducing cell apoptosis in endometrial carcinoma (Ye et al., 2014). Here, we uncovered that miR-100-5p downregulated CDC25A expression. Overexpressing CDC25A facilitated BC cell malignant progression. In addition, rescue experiment suggested that miR-100-5p hampered BC cell progression by targeting CDC25A.

In conclusion, we validated the inhibitory effect of miR-100-5p on malignant behaviors of BC cells. Besides, miR-100-5p suppresses development of BC through reducing CDC25A expression. Our study helps us to gain more awareness of role of miR-100-5p in BC, and brings additional insight into the exploration of novel targeted therapy for BC. Nevertheless, there are several limitations. For instance, it is necessary to further investigate the involvement of signaling pathways downstream of miR-100-5p/CDC25A. Besides, direct relationship of miR-100-5p and CDC25A, as well as their molecular mechanism in regulating biological behaviors are still elusive. These shortcomings need to be resolved in further studies.

## Supplemental Information

10.7717/peerj.12263/supp-1 Supplemental Information 1 Dataset

10.7717/peerj.12263/supp-2 Supplemental Information 2 Western blot

## Additional Information and Declarations

### Data Availability

The following information was supplied regarding data availability: The raw data are available as Supplemental Files.
