# Supplementary figures and images for: Role of miR-100-5p and CDC25A in breast carcinoma cells
Source: PeerJ. 2022 Jan 3;10:e12263. doi: 10.7717/peerj.12263 (PMC8734459; doi:10.7717/peerj.12263)

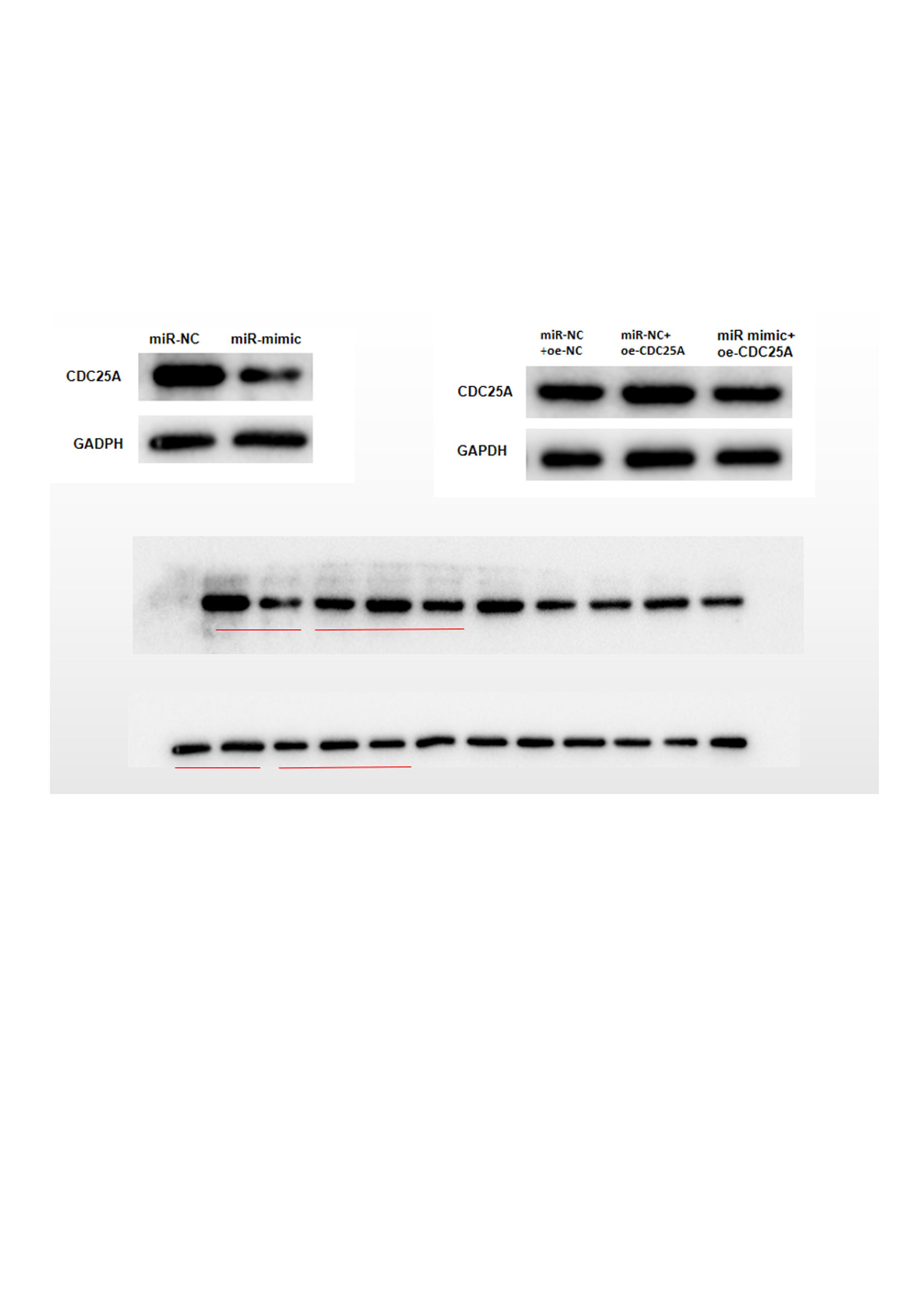

Supplement: Supplemental Information 2 [file peerj-10-12263-s002.jpg]
